# Low Carbon Sustainable Diet Choices—An Analysis of the Driving Factors behind Plant-Based Egg Purchasing Behavior

**DOI:** 10.3390/nu16162604

**Published:** 2024-08-07

**Authors:** Ping Li, I-Kai Lin, Han-Shen Chen

**Affiliations:** 1 School of Economics, Jiaxing University, Jiaxing 314001, China; lphy2010@126.com; 2 Department of Health Industry Technology Management, Chung Shan Medical University, Taichung City 40201, Taiwan; linken910428@gmail.com; 3 Department of Medical Management, Chung Shan Medical University Hospital, Taichung City 40201, Taiwan

**Keywords:** plant-based egg, low-carbon diets, sustainable development goals (SDGs), value-belief-norm theory (VBN), theory of planed behavior (TPB)

## Abstract

In the face of escalating climate change, environmental pollution, and global crises, plant-based eggs are a viable solution for environmental conservation and health promotion. This study uses an integrated framework combining value-belief-norm (VBN) theory and the Theory of Planned Behavior (TPB) to explore the factors influencing Taiwanese consumers’ inclination towards plant-based eggs. Through convenience sampling, 417 questionnaires were issued and 387 valid responses were received, indicating a 92.8% valid response rate. The analysis indicates that consumers’ biospheric and altruistic values significantly enhance their awareness of outcomes, which, coupled with their sense of responsibility, strengthens personal norms. From the TPB perspective, perceived behavioral control is identified as a critical driver of the intention to purchase plant-based eggs, with attitudes and subjective norms playing significant roles. Subjective norms positively impact personal norms, thereby influencing consumers’ behavioral intentions. The findings confirm the integrated model’s theoretical and practical validity, and offer valuable insights for businesses. By developing adept marketing strategies that consider market dynamics, this study enhances the effectiveness and value of marketing efforts by boosting consumers’ engagement with plant-based eggs. These insights contribute to advancing environmental sustainability goals.

## 1. Introduction

The burgeoning environmental footprint of the livestock industry, marked by considerable greenhouse gas emissions, has garnered increasing attention in recent years [1]. A pivotal report by the Food and Agriculture Organization (FAO) during the 28th Conference of the Parties (COP28) underlined that emissions attributable to this sector, spanning from fertilizer production to transport, culminate in approximately 6.2 billion tonnes of carbon dioxide equivalent annually, with egg production alone contributing 9% of this voluminous emission from livestock activities [2]. Concurrently, the advent of avian influenza, exacerbated by climate change, along with the heightened feed costs due to geopolitical tensions, such as the Russia-Ukraine conflict, have unearthed vulnerabilities in the global egg supply, manifesting in significant shortages since late 2021 [3,4].

Despite the contested discourse on the link between egg consumption and cardiovascular disease and related mortalities [5,6], emerging evidence posits an augmented risk associated with daily egg intake [7]. Furthermore, enhanced egg consumption has been implicated in elevated risks of cardiovascular diseases and diabetes [8], accentuating the urgency of alternative protein sources.

Amidst the escalating environmental and health problems posed by conventional livestock farming, the need for sustainable alternative protein sources has increased [9]. Individual dietary transformations are emerging as a viable solution, overshadowing reliance on governmental policies or large-scale livestock management practices [10]. In this vein, plant-based products crafted to emulate the sensorial attributes of their animal-based counterparts [11] are popular amid growing health and sustainability consciousness among consumers [12,13]. Specifically, plant-based eggs, delineated as substitutes that utilize sustainable plant resources to mimic the multifaceted aspects of traditional eggs [11], are gaining traction because of their lower environmental impact, health promotion potential, and animal welfare benefits [14]. Rondoni et al. [15] reported that plant-based eggs are healthier, more animal-friendly, and more sustainable than the traditional chicken eggs. In addition, plant-based eggs can overcome food safety concerns such as antibiotics and *Salmonella* [16]. Therefore, to achieve the Sustainable Development Goals (SDGs) of “Zero Hunger” and “Responsible Consumption and Production”, plant-based eggs are considered one of the key factors in achieving the SDGs.

The advent of food technological advancements has catalyzed the introduction of plant-based egg alternatives, as exemplified by the launch of the Just Egg by the American company Eat Just, based in San Francisco, CA, USA, in 2018, alongside Taiwan’s innovative contributions to the field through the collaborative efforts of A-Sha Foods and the National Taiwan University College of Food Science and Technology, leveraging rice protein for its hypoallergenic and nutritional advantages [17]. Despite these advancements and environmental and health motivations driving the adoption of alternative protein sources, consumer hesitancy towards plant-based foods relative to traditional animal products remains a notable barrier [18].

Additionally, according to the “Vegetarian Statistics 2024” report published by the Great Green Wall in 2024, Taiwan’s vegetarian population (including flexitarians) has reached 3 million, accounting for approximately 14% of the total population, ranking fourth globally [19]. In 2017, CNN also named Taipei in Taiwan as one of the “top ten vegetarian-friendly cities” [20]. Previous research has also indicated that vegetarians are a target group for plant-based alternatives [21]. However, plant-based eggs are still an emerging product in the Taiwanese market. Therefore, this study focuses mainly on consumers in Taiwan.

Prior studies indicate a mixed reception towards plant-based eggs across different cultural contexts, varying from health and environmental motivations [15,21,22] to sensory appeal and consumer pleasure [23]. This backdrop elucidates the dearth of research specific to the Taiwanese context, emphasizing the need to unearth the factors that resonate the most with Taiwanese consumers regarding plant-based egg adoption.

To understand individuals’ intentions and behaviors comprehensively, this study applies the value-belief-norm (VBN) theory proposed by Stern et al. [24], which highlights the relationship between personal values, new environmental paradigms, consequence awareness, and responsibility attribution. This theory has been widely used in many studies related to sustainable behaviors [25,26,27], and its applicability in Taiwan has been confirmed [28]. To further investigate individual behavioral patterns, this study utilized Ajzen’s Theory of Planned Behavior (TPB) [29]. Scholars believe that this theory can predict and explain human behavior in different settings [30], and has been successfully applied in many consumer behavior studies related to food [31,32,33]. Against this backdrop, several studies have combined TPB and VBN theories to explore various topics. These include pro-environmental behaviors [26,34], green hotels [35,36], climate change adaptation [37], natural foods [27], and green consumption [38]. However, existing research has primarily focused on general green consumption and pro-environmental behaviors, with a lack of in-depth studies on specific emerging products, such as plant-based eggs. Moreover, there is still no clear understanding of the complex relationships between various psychological and social motivations that influence the purchase of plant-based food products.

Therefore, this study aims to explore the key factors influencing Taiwanese consumers’ intention to purchase plant-based eggs by integrating TPB and VBN theories. The findings of this study contribute to understanding the reasons influencing individuals’ behavioral intentions and the purchase of plant-based eggs and provide a deeper insight into consumer behavior towards plant-based foods. Moreover, if managers in the food industry can effectively leverage the key factors driving consumers to purchase plant-based eggs, this will assist them in formulating marketing strategies and predicting market trends. Hence, this study enhances the efficacy and practical value of marketing practice. The second part of this study details the literature review and hypothesis development for VBN and TPB variables. The third part describes the framework, design, sample collection, and statistical analysis of the study. The fourth part presents the overall results of the data analysis. The fifth part discusses the results. The sixth part concludes the study and offers theoretical and practical implications.

## 2. Literature Review and Hypothesis Development

### 2.1. Value-Belief-Norm Theory

Introduced by Stern et al. [24], VBN theory presents a structured causal chain comprising five critical variables: values, new environmental paradigms, awareness of consequences, ascribed responsibility, and personal norms. Central to the VBN theory is the proposition that individual environmental behaviors are motivated and shaped by a logical sequence that begins with an individual’s fundamental values and culminates in the formation of personal norms that guide behavior. The theory articulates that when individuals perceive their core values as under threat and believe that their actions can effectively mitigate this threat, they are likely to develop a sense of responsibility to take action [39]. The following discussion elaborates on the impact of each VBN variable, drawing on existing literature to support our hypotheses.

#### 2.1.1. The Role of Value Orientation

The VBN theory indicates that value orientations influence environmental beliefs, which, in turn, affect personal norms. Park et al. [40] pointed out that values are significant predictors of behavior. Therefore, to foster personal behavior, it is important to link these actions with key values recognized and valued by individuals [41]. This study categorizes values as either biospheric or altruistic.

Biospheric and altruistic values reflect goals beyond oneself: nature and the environment, and others and society, respectively. The more people recognize these two sets of values, the more likely they are to engage in environmental actions [42,43]. Tolppanen and Kang [44] found that altruistic values only lead to a low-carbon lifestyle when combined with biospheric values, but not when combined with hedonistic values. Similarly, multiple studies have shown that individuals with biospheric and altruistic values are more inclined towards behaviors that benefit the environment than are those with hedonistic or egoistic values [45,46].

#### 2.1.2. Environmental Beliefs and Awareness

Within VBN theory, environmental attitudes are conceptualized as beliefs, incorporating the notion of a new environmental paradigm [24]. These beliefs encompass awareness of the consequences (AC) of environmental issues and play a pivotal role in shaping environmental responsibility and behaviors. Hornsey and Fielding [47] indicate that most people still doubt the facts of climate change; therefore, changing people’s beliefs and cognition is crucial. A cross-national study showed that biospheric values are an important prerequisite for an individual’s awareness of the risks and consequences of climate change [48]. Research by Çakır Yıldırım and Karaarslan Semiz [49] pointed out that biospheric altruistic values have a positive and significant impact on consequence awareness, which also mediates the relationship between biospheric altruistic values and ascription of responsibility. In summary, this study proposes the following hypotheses:

**H1a.**  *Biospheric values have a significant and positive impact on the awareness of consequences.*

**H1b.**  *Altruistic values have a significant and positive impact on the awareness of consequences.*

Ascription of responsibility (AR) refers to the possibility that an individual may be held accountable for the negative consequences of certain situations [50]. Abdallah et al. [51] stated that humans are the drivers of naturally occurring climate changes, and people should take responsibility for reducing environmental change. Carfora et al. [27] on natural foods found that consumers’ cognition of the consequences of purchasing natural foods affects their views on the ascription of responsibility, which in turn affects their behavioral intention to purchase natural foods. The research of Al Mamun et al. [52] on managing solid waste also indicated that participants’ awareness of consequences significantly and positively affects their ascription of responsibility. In summary, this study proposes the following hypothesis:

**H2.**  *Awareness of the consequences has a significant and positive impact on ascription of responsibility.*

#### 2.1.3. Personal Norms and Environmental Behavior

Personal norms are seen as an extension of self-value concepts, referring to the externalization of duties and moral awareness, and are considered a self-regulatory consciousness that drives environmental behavior [53]. Esfandiar et al. [54] found that personal norms play a significant role in predicting pro-environmental behavior, with the strongest association between consequence awareness and personal norms. Wu et al. [55] pointed out that responsibility attribution has a positive impact on personal norms, while the activation of personal norms by consequence awareness plays a decisive role. Based on the above, this study proposes the following hypotheses:

**H3a.**  *Responsibility attribution has a significant and positive impact on personal norms.*

**H3b.**  *Consequence awareness has a significant and positive impact on personal norms.*

### 2.2. Theory of Planned Behavior

The TPB posits that an individual’s intention to engage in a particular behavior is chiefly influenced by three determinants: attitude towards the behavior, subjective norms, and perceived behavioral control [29]. Positive attitudes and subjective norms, in conjunction with a strong sense of behavioral control, tend to bolster the intention to perform an associated action [56]. Research by Alam et al. [57] empirically supports TPB, indicating that attitudes, subjective norms, and perceived behavioral control significantly and positively impact sustainable food consumption intentions. This finding is corroborated by studies by Khan et al. [58] and Li et al. [59], which affirm the crucial role these three components play in fostering green behavioral intentions. This study aims to delve deeper into the effects of these and other factors on the TPB framework, as elaborated in the following subsections.

#### 2.2.1. Behavioral Intention (BI)

Behavioral Intention refers to an individual’s predilection and commitment towards a particular action, essentially representing the psychological readiness to engage in that action within the context of behavioral choices [29]. A robust BI serves as a solid predictor of actual behavior, effectively bridging the intention-behavior gap [60]. Le and Nguyen [61] suggested that personal norms influence organic food purchase intentions and serve as mediating variables in their proposed model. Similarly, Al Mamun et al. [52] posited that personal norms play a predictive role in solid waste management intentions with the aim of reducing environmental impact. Based on these insights, we propose the following hypothesis:

**H4.**  *Personal norms have a significant and positive effect on behavioral intention.*

#### 2.2.2. Attitude (AT)

In the realm of TPB, an individual’s attitude encompasses their enduring evaluative stance towards specific entities, predicting behavioral predispositions [29]. Numerous studies focusing on green products have demonstrated a positive relationship between pro-environmental attitudes and purchase intentions, aligning with TPB [62,63]. Liu et al. [64] underscored the importance of attitudes in predicting purchase intentions. Therefore, this study proposes the following hypothesis:

**H5.**  *Attitude has a significant and positive effect on behavioral intention.*

#### 2.2.3. Subjective Norm (SN)

Subjective norm is conceptualized as the perceived pressure from key social entities (e.g., family, friends, and colleagues) to perform or abstain from a certain behavior [29]. It also reflects the broader societal and environmental forces that shape an individual’s behavioral intentions. While numerous studies have found positive correlations between subjective norms and behavioral intentions [65,66], Kamalanon et al. [67] suggest that the relationship may not always be significant regarding green product purchase intentions. This discrepancy highlights the need for further investigation into the factors that bridge subjective norms and intentions. Empirical studies by Le and Nguyen [61] and Wang et al. [36] indicated that subjective norms positively influence personal norms. Accordingly, this study proposes the following hypotheses:

**H6.**  *Subjective norms have a significant and positive impact on behavioral intention.*

**H7.**  *Subjective norms have a significant and positive impact on personal norms.*

#### 2.2.4. Perceived Behavioral Control (PBC)

Perceived behavioral control refers to an individual’s perception of their ability to control the resources and opportunities needed when engaging in a specific behavior [29]. When consumers believe that they can control these factors, their likelihood of engaging in green purchasing behavior also increases [65]. Research by Xu et al. [68] also indicated that when consumers find it easier to access green furniture, they are more willing to purchase it. Dorce et al. [32] suggested that enhancing consumers’ perceived ability to buy organic vegetables contributes to an increase in their willingness to purchase. Lim and An [69] confirmed that perceived behavioral control is an important factor in predicting behavioral intentions. Based on the above, this study proposes the following hypothesis:

**H8.**  *Perceived behavioral control has a significant and positive impact on behavioral intention.*

In synthesizing the literature review, this study integrates insights from both VBN theory and TPB, establishing a comprehensive framework for understanding environmentally responsible behaviors. The hypotheses developed (H1a to H8) aimed to test theoretical propositions within this integrated framework, focusing on the interplay between personal norms and behavioral intentions as influenced by attitudes, subjective norms, and perceived behavioral control.

## 3. Research Methodology

### 3.1. Research Framework

**Figure 1:**
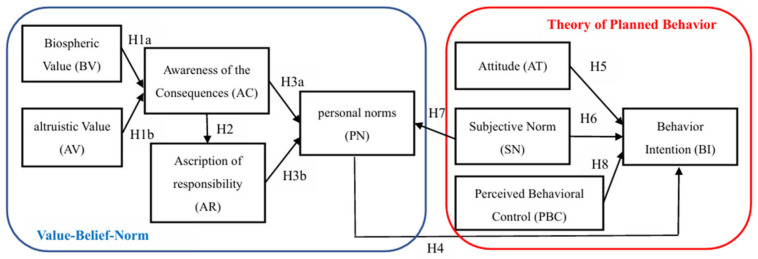
Research framework diagram.

In this study, the theoretical integration of the VBN theory of responsible environmental behavior, as proposed by Stern et al. [24], and the TPB by Ajzen [29], forms the cornerstone of the research framework. This study aimed to elucidate Taiwanese consumers’ behavioral intentions towards the adoption of plant-based eggs. The constructed framework, depicted in Figure 1, posits that the interplay between environmental values, beliefs, norms (as per VBN theory), and TPB components (attitude, subjective norms, and perceived behavioral control) collectively influence consumers’ behavioral intentions towards plant-based eggs. This integrated approach is innovative in its application and is expected to offer a nuanced understanding of consumer behavior within this context.

### 3.2. Research Design

The questionnaire was meticulously constructed in three parts to capture the multifaceted influences on Taiwanese consumers’ intentions towards plant-based eggs.

Part One operationalizes VBN theory by adapting items from seminal studies by Chen [70], Carfora et al. [27], Youn et al. [71], Li et al. [72], and Bidwell [73]. It measures biospheric and altruistic values, awareness of consequences, responsibility attribution, and personal norms, using 15 carefully crafted questions.

Part Two draws upon the TPB framework, with items adjusted from studies by Pang et al. [74], Carfora et al. [27], Tan et al. [75], Roh et al. [76], Aliedan et al. [77], Alam et al. [57], and Wang et al. [78]. It assesses attitudes, subjective norms, perceived behavioral control, and behavioral intentions, and spans 12 questions. Both parts employed a seven-point Likert scale to enhance the precision of respondent evaluations.

Part Three gathers demographic information (gender, age, education level, and monthly income), which is essential for analyzing the data in relation to various consumer segments.

Prior to distribution, the questionnaire underwent a rigorous expert review process to ensure clarity, relevance, and comprehensive coverage of the constructs. This process was crucial for refining the questions to avoid respondent misunderstanding or non-reflective answers. Suggestions for refinement from the expert panel were integrated to solidify the questionnaire’s validity.

A pilot study of 63 participants yielded a total of 55 valid responses. Through item analysis and a reliability test, the questionnaire constructs were confirmed to be both reliable and valid, making it ready for the main survey distribution.

The questionnaires were meticulously screened upon collection. Those with incomplete answers or patterned responses were dismissed to maintain the data integrity. This rigorous process ensured that the subsequent analysis was grounded in accurate and representative data, thus bolstering the reliability of the findings of the study.

### 3.3. Sample and Data Collection

The advent of the digital era, marked by the widespread accessibility and utility of the internet, has significantly influenced social science research methodologies. Traditional paper-based approaches to questionnaire distribution have gradually ceded ground to digital platforms, leveraging the internet and various social media channels as primary vehicles for data collection. This transition reflects not only an evolving technological landscapes, but also changing researcher and participant preferences. According to Sammut et al. [79], the pivot to online questionnaires initially faced with lower response rates offers compensatory benefits. These include the potential for higher completion rates, fostered by pre-notification strategies via email, and the strategic design of concise, participant-friendly questionnaires (limiting the duration to approximately 10 min).

In this study, questionnaires were disseminated via various online platforms, including personal accounts on Facebook, Instagram, and Line, leveraging natural networks within these communities to enhance participation. Adherence to ethical standards was paramount, with explicit clarification of the research objectives provided at the outset of the questionnaire, and commitment to anonymity to assuage any concerns regarding privacy. Given the non-interactive, non-interventional design of the study coupled with the absence of identifiable personal information, the prerequisite for prior ethical review was deemed unnecessary.

This study employed SEM to address the multifaceted relationships between variables and adequately explore latent constructs. SEM is distinguished by its ability to simultaneously process multiple interdependencies across independent and dependent variables [80], thereby offering a robust framework for hypothesis testing within the scope of this study. Echoing the guidelines proposed by Wu [81], the determination of an appropriate sample size for SEM requires a ratio between 10:1 and 15:1 with respect to the number of items. Accordingly, with 27 items delineating the study, the optimal sample size was projected to be between 270 and 405 participants.

The formal execution of the questionnaire was carried out over the period extending from April to June 2024. This phase culminated with 417 responses. After meticulous screening to exclude 30 inadmissible submissions, 387 questionnaires were validated for the analysis.

### 3.4. Methods of Data Analysis

In this investigation, a methodologically sophisticated approach was meticulously adopted to scrutinize the amassed data, with quantitative research methodologies serving as cornerstones. Data were diligently procured through an expansive questionnaire survey that was meticulously engineered to encapsulate a comprehensive spectrum of variables directly relevant to the articulated research aims. This study harnessed the advantages of cutting-edge statistical software tools to ensure exhaustive and methodical analysis of acquired data. Specifically, IBM SPSS Statistics 27 was deployed to execute general statistical analyses, whereas AMOS version 26 was selected for its advanced capabilities in conducting SEM, thereby providing a robust platform for detailed data examination.

The arsenal of statistical analysis methods deployed in this study was multifaceted, encompassing descriptive statistics (including frequency distribution tables, percentages, means, and standard deviations) to provide a foundational understanding of the dataset. Reliability and validity analyses were conducted to ascertain the consistency and accuracy of the instruments used to capture the data. Furthermore, SEM employing maximum likelihood estimation was employed as a pivotal analytical tool to unravel the causal interconnections postulated in the theoretical framework and rigorously assess the overall congruence of the hypothesized model with empirical data. This multifaceted analytical approach was instrumental in validating the research hypotheses proposed in this study, thereby elucidating relationships and dynamics at play.

## 4. Analysis and Results

### 4.1. Demographic Analysis

A total of 387 questionnaires were collected for this study, with 51.2% male and 48.8% female. This is roughly in line with Taiwan’s gender ratio (49:50) [82]. The age distribution showed that the largest group is aged 51–60 years (37.2%), followed by 21–30 years (20.9%), 31–40 years (18.6%), and 41–50 years (17.8%), indicating that the participants were primarily concentrated in the middle-aged group (94.5%).

Regarding educational level, 76.7% of the participants held a college degree or higher, compared to 49.7% of the population in Taiwan with a college degree or above [83]. This finding suggests that our sample consists of a higher proportion of highly educated individuals. The distribution of pre-tax personal monthly income shows that the largest income group among participants is NTD 40,001–60,000 (25.6%), followed by NTD 60,001–80,000 (20.2%) and NTD 80,001–100,000 (20.2%). Compared to the average monthly income in Taiwan of NTD 48,422 [84], most participants had a higher income level.

In terms of dietary habits, 334 participants were omnivorous (86.3%) and 53 were flexitarians (13.7%), which aligns with the percentage of vegetarians in Taiwan (14%) [19]. Table 1 systematically categorizes the detailed demographic information of the participants, serving as the demographic basis for the subsequent predictive analysis.

### 4.2. Measurement Model: Reliability and Validity

To ensure the empirical robustness of this investigation, we deployed a bifurcated analytical methodology. The inaugural phase utilized Confirmatory Factor Analysis (CFA), an integral component of SEM aimed at scrutinizing the nexus between the observable variables and their latent counterparts (factors). In this context, the essence of CFA is to ascertain the efficacy of latent variables that accurately manifest through observable variables. Typically, CFA plays a pivotal role in assessing psychometrics, determining construct validity, examining test method effects, and verifying model group invariance. Owing to the adoption of survey instruments conceived by previous researchers, the necessity for CFA in this study is paramount, thereby testing its suitability across the demographic landscape of this research.

The analytical spectrum of this study spans nine distinct sub-dimensions: Biospheric Values, Altruistic Values, Awareness of Consequences, Attribution of Responsibility, Personal Norms, Attitudes, Subjective Norms, Perceived Behavioral Control, and Behavioral Intentions. Sequential CFA across each dimension constituted the initial procedural step, which involved the exclusion of items with factor loadings below the threshold of 0.5. This purging process, inferred from the preliminary analysis outcomes, underpinned the subsequent iterations of the CFA aimed at assessing the Root Mean Square Error of Approximation (RMSEA) for the enumerated sub-dimensions. An RMSEA exceeding the benchmark of 0.08 typifies suboptimal model fit, necessitating iterative model refinements predicated upon the modification index (MI) deletion guidelines, until the attainment of an RMSEA below 0.08, or the emergence of a saturated model configuration.

Subsequent to the delineation of the sub-dimensional items, comprehensive evaluations encompassing composite reliability (CR) and convergent validity for each dimensional facet were executed expeditiously. The CR metric, encapsulating the reliability quotient of all measurement variables, varies between 0 and 1, with higher values indicative of elevated “true variance” representation within the total variance, essentially denoting superior internal consistency. Fornell and Larcker [85] posited that the CR values of latent variables should surpass 0.60, which is the benchmark for reliability. The diagnosis of the latent variables’ convergent validity primarily employs the Average Variance Extracted (AVE) metric, with thresholds exceeding 0.50, as recommended by Fornell and Larcker [85], alongside Bagozzi and Yi [86], which is indicative of robust convergent validity.

In this study, CR values ranged from 0.885 to 0.935 across the dimensions, indicating high internal consistency of the scale. Similarly, AVE values span from 0.720 to 0.828, surpassing the 0.50 benchmark and affirming strong convergent validity. Standardized regression weights for all items varied from 0.764 to 0.948, with all t-values exceeding the critical value of 1.96, denoting significant loadings. These metrics, as detailed in Table 2, confirm that the dimensions of the questionnaire satisfactorily meet the convergent validity criteria, underlining the internal coherence of the measurement model.

Further, discriminant validity was assayed by exploring correlations between divergent constructs, postulating that low correlation levels would signify distinct conceptual identities. According to Hair et al. [87], the correlation coefficient between the two concepts should be less than the square root of the AVE for each concept. Table 3 shows a comparison of all construct correlation coefficients and the square root of the AVE for this study. The square root values of each construct’s AVE are greater than the correlation coefficient between the two constructs, meeting the standard suggested by Hair et al. [87] and indicating discriminant validity between the constructs of this study. Based on the evaluation results of the above measurement model, it can be seen that the measurement model of this study has good internal and external quality.

To ensure that our study was not affected by common method bias, we used Harman’s single-factor test to assess and report any potential biases and examine whether the common method variance among the study variables reached problematic levels, thereby impacting the research results (Table 4). The explained variance of the first factor in our study was 46.446%, which did not exceed 50%, indicating that common method bias was not a severe issue [88].

### 4.3. Model Fit Test

In this study, the adequacy of the structural model was assessed using the maximum likelihood estimation (MLE) method, a widely recognized approach for examining theoretical model structures within the social sciences. As part of this evaluation, various fit indices were meticulously analyzed to gauge the model’s conformity with the observed data. As shown in Table 5, these indices include the chi-square to degrees of freedom ratio (*χ*^2^/*df* = 2.632), Root Mean Square Residual (RMR = 0.042), Root Mean Square Error of Approximation (RMSEA = 0.072), Adjusted Goodness of Fit Index (AGFI = 0.812), Normed Fit Index (NFI = 0.902), Comparative Fit Index (CFI = 0.911), and Incremental Fit Index (IFI = 0.913).

Each of these indices satisfies the conventional threshold values appropriate for model fit assessment, thereby substantiating the commendable fit of the structural model to the empirical data. Specifically, the *χ*^2^/*df* ratio, which elucidates the simplicity and parsimony of the model, fell well below the accepted upper limit of 3. RMSEA and RMR values, pivotal in signifying the residual variances, were also within the recommended benchmarks, indicating a minimal discrepancy between the hypothesized model and actual data. Correspondingly, the AGFI, NFI, CFI, and IFI indices surpassed the generally accepted criterion of 0.8, confirming the robustness of the model and its predictive accuracy.

### 4.4. Overall Model Path Analysis

**Figure 2:**
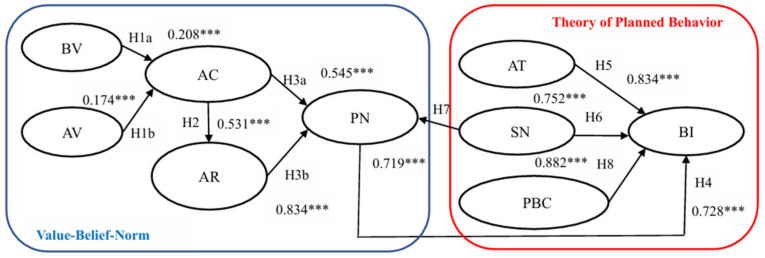
Structural equation modeling diagram. Note: *** *p* < 0.001.

Employing SEM, this study comprehensively examined the hypothesized relationships delineated within the proposed theoretical framework. Notably, the structural model analysis, visually represented in Figure 2, afforded a systematic investigation of the dynamics between the variables. The path coefficients, which indicate the strength and direction of the relationships among the variables, reveal significant insights.

H1a and H1b propose that both biospheric (β = 0.208, *p* < 0.001) and altruistic values (β = 0.174, *p* < 0.001) exert a significantly positive effect on outcome awareness, affirming the foundational role of core values in shaping environmental consciousness.

H2 elucidates the profound influence of outcome awareness on responsibility attribution (β = 0.531, *p* < 0.001), illustrating a critical psychological mechanism where recognizing environmental outcomes heightens perceived personal responsibility.

The effects of outcome awareness (β = 0.545, *p* < 0.001) and responsibility attribution (β = 0.834, *p* < 0.001) on personal norms articulated in H3a and H3b, respectively, suggest a substantial causal pathway through which environmental concerns translate into personal ethical standards.

H4 to H6 and H8 delineated the significant positive influences of personal norms (β = 0.728, *p* < 0.001), attitudes (β = 0.834, *p* < 0.001), subjective norms (β = 0.752, *p* < 0.001), and perceived behavioral control (β = 0.882, *p* < 0.001) on behavioral intentions. These findings underscore the multifaceted motivational underpinnings of environmentally responsible behaviors.

Lastly, H7’s confirmation (β = 0.719, *p* < 0.001) that subjective norms positively impact personal norms reinforces the interplay between societal expectations and individual moral guidelines.

In summary, the path analysis and hypothesis testing results, encapsulated in Table 6, unequivocally validated the significant and meaningful relationships posited in H1a through H8. The findings provide compelling empirical support for the theoretical construct, suggesting that intrinsic values, awareness, responsibility attribution, and personal norms interact intricately to foster pro-environmental behavioral intentions.

## 5. Discussion

This research employs a synthesized framework combining the VBN theory and TPB to examine the determinants of Taiwanese consumers’ intentions towards purchasing plant-based eggs. The integration of these theories provides a comprehensive perspective that acknowledges both ethical values and pragmatic considerations in the consumer decision-making processes.

### 5.1. The Influence of Biospheric and Altruistic Values

Our findings reveal that both biospheric and altruistic values exert a positive and substantial impact on outcome awareness, echoing the research conducted by Martin [48] and Çakır Yıldırım and Karaarslan Semiz [49]. Notably, biospheric values demonstrated a more pronounced effect (β = 0.208), which can be attributed to the global and pressing nature of environmental issues, such as climate change and biodiversity loss. Owing to their perceived urgency and irreversibility, these issues likely engender a heightened sense of concern and perceived risk among individuals [89]. This observation aligns with Martin’s [48] findings that in contexts characterized by higher wealth and individualism, the significance of biospheric values and perceived personal risk is amplified. This finding supports the notion that societal factors play a critical role in shaping environmental values and perceptions.

### 5.2. Outcome Awareness as a Mediator

The significant positive linkage between outcome awareness and attribution of responsibility corroborates Carfora et al.’s [27] assertion that consequence perception plays a pivotal role in fostering a sense of responsibility towards purchasing eco-friendly products. This suggests that outcome awareness can effectively translate external environmental concerns into a personal sense of accountability, in harmony with VBN theory’s postulations [24]. The model further extends the analysis by elucidating the transformative potential of outcome awareness in awakening personal morality and promoting adherence to ethical norms, which have been less explored in prior research.

Furthermore, in terms of H3a and H3b, most previous studies have only explored the relationship between attribution of responsibility and personal norms [35,40,49,55,90], with less attention paid to the relationship between outcome awareness and personal norms. However, we believe that outcome awareness can awaken an individual’s inner sense of morality, prompting them to adhere to higher standards of behavior and ethical norms. The results also showed that both outcome awareness and attribution of responsibility had positive and significant effects on personal norms. This finding is consistent with that of previous research [54,55]. This study also found that the higher an individual’s personal norms, the higher their behavioral intention to purchase plant-based eggs (β = 0.728). This implies that when individuals are more inclined to follow internal moral demands and norms, they also have a higher intention to purchase eco-friendly products such as plant-based eggs. This finding is consistent with research on organic food by Le and Nguyen [61] and solid waste management by Al Mamun et al. [52].

### 5.3. Attitudes, Subjective Norms, and Perceived Behavioral Control

On the other hand, the research validated hypotheses H5, H6, and H8, showing that attitudes, subjective norms, and perceived behavioral control have a positive and significant impact on the intention to purchase plant eggs. This result is consistent with the findings of most studies that have adopted the TPB [63,91]. This aligns with Ajzen’s [29] proposal in the TPB that behavioral intentions are driven by attitudes, subjective norms, and perceived behavioral controls.

Notably, this study found perceived behavioral control to be a key factor influencing behavioral intentions (β = 0.882), possibly because the sample collected mostly consisted of individuals with higher education levels and income. We believe that these groups may thus have higher environmental and health consciousness, and are capable of choosing products such as plant eggs on their own. This finding is in line with Tianyu and Meng [92], who found that a higher level of education increases an individual’s willingness to pay for environmental protection. Philippssen et al. [93] found that educational level and income were positively related to environmental awareness.

This study confirmed that subjective norms have a significant positive effect on personal norms. This means that external social pressures and expectations can influence an individual’s internal norms and decision-making and make them more likely to internalize them as their own values and behavioral standards. This corroborates the study by Wang et al. [36], which indicated that subjective norms are internalized through personal norms and indirectly or directly affect the intention to purchase green hotels.

In particular, the impact of subjective norms on behavioral intentions is higher than that of personal norms (β = 0.752), indicating that Taiwanese consumers are more inclined to seek advice and follow others’ references when intending to purchase plant-based foods, rather than relying solely on their own values and personal norms for decision-making. This confirms the significant influence of social pressure and the expectations of others on behavioral decision making [94]. This phenomenon may be closely related to Taiwan’s multicultural background and the sociocultural characteristics of placing a high value on interpersonal relationships.

### 5.4. Contributions and Implications

This study contributes to the extant literature by bridging the VBN theory and TPB, thereby offering a nuanced understanding of the moral and practical considerations that underpin consumer behavior in the context of sustainable consumption. This underscores the complexity of ethical decision-making, where both intrinsic values and perceived societal expectations coalesce to shape consumer intentions towards eco-friendly products, such as plant-based eggs.

These findings have significant implications for marketers, policymakers, and advocates of social change. Tailoring communication strategies to highlight the urgent environmental benefits of sustainable consumption while fostering a supportive social milieu that values sustainability could enhance consumer engagement with eco-friendly products. Additionally, enabling conditions that boost perceived behavioral control through accessibility and affordability further encourage sustainable consumption practices.

In conclusion, this discussion has enriched our understanding of the multifaceted influences on eco-friendly consumer behavior, providing a robust foundation for future inquiries and interventions aimed at promoting sustainability in consumer choices.

## 6. Conclusions and Recommendations

### 6.1. Research Conclusions

To understand the factors affecting consumers’ intention to purchase plant-based eggs, this study integrated TPB and the VBN theory to form a comprehensive framework for a deeper exploration of consumer behavior. Several important results were obtained from this study.

First, it was found that the intention to purchase plant-based eggs is closely related to the variables of TPB, with perceived behavioral control being the most significant predictor of Taiwanese consumers’ purchase intention, followed by attitude and subjective norms. If businesses can understand the driving factors behind consumer intentions, this would greatly contribute to the formulation of marketing strategies.

Second, the study verified the linear relationship between values, outcome aware-ness, responsibility attribution, and personal norms within the VBN theory. The results show that biospheric values have a greater impact on outcome awareness than altruistic values. Responsibility attribution had a greater impact on personal norms than outcome awareness, and outcome awareness positively affected responsibility attribution.

Third, the results and model hypotheses of this study are consistent, which helps advance the study of consumer behavior in plant-based foods and proves that this integrated model can be applied in food-related research.

### 6.2. Management Recommendations

In management practice, businesses can develop marketing strategies that target values and ethical beliefs, influencing and reshaping consumers’ existing values (e.g., biospheric values) to increase their likelihood of purchasing plant-based eggs.

This study also indicates that enhancing consumers’ perceived behavioral control helps boost their purchasing intentions. Therefore, we recommend that businesses leverage digital media such as the Internet to expand their marketing channels, reduce barriers to purchasing plant-based eggs, and enhance the convenience of buying plant-based eggs in regular stores.

Additionally, organizing promotional events or cooking workshops for plant-based egg products at community centers, schools, supermarkets, etc., to introduce consumers to the nutritional value, health benefits, and cooking methods of plant-based eggs can help them become more familiar with the product. These are all directions that managers can focus on in the future.

Moreover, the government should incorporate plant-based eggs into healthy dietary plans. By providing production subsidies and marketing incentives, lowering market prices, and increasing product affordability, the government can support businesses in expanding production and sales of plant-based eggs.

### 6.3. Research Limitations and Future Research Directions

Although this study has elucidated the key aspects of consumer intentions towards plant-based eggs, it has several limitations.

First, it focuses primarily on Taiwanese consumers and does not discuss specific groups (e.g., egg buyers). Based on the results of this study, future surveys should extend beyond the Taiwanese context to cover different population groups, thereby enriching the generalizability and depth of understanding of consumer intentions. Such a research design can provide a more specific understanding of the needs and behavioral patterns of particular groups.

In this study, the participants were from various regions of Taiwan, including urban and rural areas. However, consumer behavior in urban and rural areas may differ [95,96]. Therefore, future research should consider further segmenting the proportion of urban and rural areas within the sample to gain a more comprehensive understanding of behavioral differences in these regions.

Additionally, Taiwan has a high proportion of vegetarians, who are among the main consumers of plant-based food. Therefore, future research could distinguish between vegetarians and non-vegetarians, and explore the differences in purchasing behavior for plant-based eggs between these two groups. This differentiation would help develop targeted marketing strategies and understand the factors influencing purchasing decisions for different groups.

Furthermore, our study used convenience sampling, which, according to Zickar et al. [97] and Emerson [98], has limitations such as potential data quality issues and lack of generalizability. Therefore, future research should consider using more representative sampling methods, such as random or stratified sampling, to improve the representativeness of the sample and external validity of the research findings.

Finally, as a novel and environmentally sustainable product, future research should incorporate variables such as food neophobia, health consciousness, and price sensitivity, to make the research framework more comprehensive.

By advancing these imperative recommendations and acknowledging the limitations, future endeavors can build on this research foundation, propelling scholarly and practical understanding of consumer behavior in the burgeoning domain of plant-based foods.

## Figures and Tables

**Table 1:** Demographic analysis.

N = 387	Item	Sample Size	Percentage (%)
Gender	Male	198	51.2
	Female	189	48.8
Age	20 years and below	6	1.6
	21–30 years	81	20.9
	31–40 years	72	18.6
	41–50 years	69	17.8
	51–60 years	144	37.2
	60 years and above	15	3.9
Education Level	High school/vocational or below	90	23.3
	College/university	189	48.8
	Master’s or above	108	27.9
Personal pre-tax monthly income	Less than NTD 20,000 (USD 660) (inclusive)	63	16.3
	NTD 20,001–40,000 (USD 660–1320)	48	12.4
	NTD 40,001–60,000 (USD 1320–1980)	99	25.6
	NTD 60,001–80,000 (USD 1980–2640)	78	20.2
	NTD 80,001–100,000 (USD 2640–3300)	78	20.2
	Above NTD 100,001 (USD 3300)	21	5.4
Occupation	Student	66	17.1
	Army, civil service, and education	150	38.8
	Service industry	84	21.7
	Freelance	27	7.0
	Traditional manufacturing	15	3.9
	Specialized occupation (e.g., doctor and lawyer)	15	3.9
	Other (e.g., temporary workers, homemakers and retirees)	30	7.8
Dietary habits	Omnivorous	334	86.3
	Flexitarian	53	13.7

**Table 2:** Results related to factor loading, reliability, and validity.

Variables/Items	Standardized Factor Loading	CR	AVE	Cronbach’s α
Biospheric Values (BV)		0.896	0.742	0.821
1. I can feel an emotional connection with nature	0.815 ***			
2. I believe the environment can be protected and preserved	0.883 ***			
3. I think humans can live in harmony with other species	0.884 ***			
Altruistic Values (AV)		0.900	0.751	0.801
4. I pursue a world of peace, without wars and conflicts	0.904 ***			
5. I support social justice, correcting injustices, and caring for the vulnerable	0.923 ***			
6. I believe in equality for all, with equal opportunities	0.764 ***			
Awareness of Consequences (AC)		0.885	0.720	0.802
7. I think the modern livestock production system causes environmental pollution, climate change, and depletion of natural resources	0.858 ***			
8. I believe the type of food we choose to eat can have a positive or negative impact on the environment	0.850 ***			
9. I think choosing to buy plant-based eggs is an action that can help improve the Earth’s environment	0.837 ***			
Attribution of Responsibility (AR)		0.898	0.747	0.824
10. I believe that, as citizens, buying plant-based eggs is a way for us to share the responsibility of protecting the environment	0.789 ***			
11. I think every consumer should take responsibility for the environmental degradation caused by their choice of food and consumption behavior	0.870 ***			
12. I believe every consumer should bear some responsibility for the environmental problems caused by modern livestock farming	0.928 ***			
Personal Norms (PN)		0.903	0.758	0.839
13. I believe I have a duty to choose plant-based eggs to make the environment more sustainable	0.913 ***			
14. I think choosing food in an environmentally friendly way is crucial for every consumer	0.801 ***			
15. I believe that for the sake of environmental protection, we should prioritize buying plant-based eggs over animal eggs	0.893 ***			
Attitudes (AT)		0.935	0.828	0.893
16. I believe from a health perspective, plant-based eggs are superior to animal eggs	0.855 ***			
17. My attitude towards buying plant-based eggs is positive and active	0.925 ***			
18. I believe purchasing plant-based eggs is a responsible and wise decision	0.948 ***			
Subjective Norms (SN)		0.875	0.802	0.924
19. My family thinks I should choose to buy plant-based eggs to protect the environment	0.877 ***			
20. I am influenced by news, newspapers, and magazines to choose plant-based eggs	0.915 ***			
21. The positive feedback from my friends/colleagues about plant-based eggs makes me more inclined to buy them	0.894 ***			
Perceived Behavioral Control (PBC)		0.905	0.760	0.841
22. If given the chance, I would choose to buy plant-based eggs	0.872 ***			
23. I have enough resources, financial means, and time to buy plant-based eggs	0.836 ***			
24. I have sufficient knowledge to purchase and choose plant-based eggs	0.906 ***			
Behavioral Intention (BI)		0.935	0.827	0.896
25. Considering the lower environmental pollution caused by plant-based eggs, I will consider buying them	0.885 ***			
26. If I happen to find plant-based eggs in a store, I will choose to buy them	0.919 ***			
27. I plan to actively seek out and buy plant-based eggs in stores	0.924 ***			

Note 1: CR = composite reliability; AVE = average variance extracted. Note 2: *** *p* < 0.001.

**Table 3:** Discriminant validity test.

	Mean	Standard Deviation	1.	2.	3.	4.	5.	6.	7.	8.	9.
1. BV	5.477	1.235	**0.861**								
2. AV	6.235	0.927	0.500 **	**0.867**							
3. AC	5.284	1.017	0.208 **	0.174 **	**0.848**						
4. AR	5.507	1.073	0.584 **	0.362 **	0.531 **	**0.864**					
5. PN	5.215	1.071	0.559 **	0.310 **	0.545 **	0.834 **	**0.870**				
6. AT	4.749	1.222	0.396 **	0.223 **	0.481 **	0.659 **	0.799 **	**0.910**			
7. SN	4.744	1.191	0.456 **	0.264 **	0.344 **	0.611 **	0.719 **	0.843 **	**0.895**		
8. PBC	4.767	1.223	0.161 **	0.182 **	0.445 **	0.470 **	0.622 **	0.791 **	0.700 **	**0.872**	
9. BI	4.739	1.257	0.313 **	0.207 **	0.452 **	0.549 **	0.728 **	0.834 **	0.752 **	0.862 **	**0.909**

Note 1: The values in bold font are the square roots of the AVE; non-diagonal numbers represent the correlation coefficients of each dimension. Note 2: BV = biospheric value, AV = altruistic value, AC = awareness of the consequences, AR = ascription of responsibility, PN = personal norms, AT = attitude, SN = subjective norm, PBC = perceived behavioral control, BI = behavioral intention. Note 3: ** *p* < 0.01

**Table 4:** Common method bias test.

Component	Initial Eigenvalues		
	Total	% of Variance	Cumulative %
1	12.540	46.446	46.446
2	3.233	11.975	58.420
3	1.913	7.086	65.506
4	1.594	5.906	71.412
5	0.942	3.489	74.900
6	0.891	3.298	78.199
7	0.685	2.538	80.737
8	0.518	1.918	82.655
9	0.501	1.856	84.510
10	0.467	1.730	86.241
11	0.455	1.686	87.927
12	0.414	1.534	89.461
13	0.361	1.337	90.798
14	0.330	1.222	92.019
15	0.310	1.147	93.166
16	0.274	1.016	94.182
17	0.247	0.915	95.096
18	0.222	0.822	95.919
19	0.200	0.739	96.658
20	0.171	0.634	97.292
21	0.162	0.600	97.892
22	0.130	0.480	98.372
23	0.121	0.448	98.820
24	0.105	0.390	99.210
25	0.087	0.321	99.531
26	0.070	0.259	99.790
27	0.057	0.210	100.000

**Table 5:** Analysis of fit indices.

Statistic	Recommended Value	Obtained Value	Meets Standard
$x^{2}$/*df*	<3	2.632	Yes
RMR	<0.05	0.042	Yes
RMSEA	≤0.05 (marginal fit)	0.072	Good fit
	0.05–0.08 (good fit)		
	0.08–0.10 (moderate fit)		
	>0.10 (poor fit)		
AGFI	>0.8	0.812	Yes
NFI	>0.9	0.902	Yes
CFI	>0.9	0.911	Yes
IFI	>0.9	0.913	Yes

Note: root mean square residual (RMR), root mean square error of approximation (RMSEA), adjusted goodness of fit index (AGFI), normed-fit-index (NFI), comparative fit index (CFI), incremental fit index (IFI).

**Table 6:** Results of the path analysis and confirmation of hypotheses.

Hypothesized Paths	Unstandardized Coefficient	S.E.	*p*	Standardized Coefficients	Verification Results
H1a: BV → AC	0.171	0.041	<0.001	0.208 ***	Supported
H1b: AV → AC	0.191	0.055	<0.001	0.174 ***	Supported
H2: AC → AR	0.560	0.046	<0.001	0.531 ***	Supported
H3a: AC → PN	0.574	0.045	<0.001	0.545 ***	Supported
H3b: AR → PN	0.832	0.028	<0.001	0.834 ***	Supported
H4: PN → BI	0.854	0.041	<0.001	0.728 ***	Supported
H5: AT → BI	0.858	0.029	<0.001	0.834 ***	Supported
H6: SN → BI	0.794	0.035	<0.001	0.752 ***	Supported
H7: SN → PN	0.646	0.032	<0.001	0.719 ***	Supported
H8: PBC → BI	0.907	0.025	<0.001	0.882 ***	Supported

Note 1: BV = biospheric value, AV = altruistic value, AC = awareness of the consequences, AR = ascription of responsibility, PN = personal norms, AT = attitude, SN = subjective norm, PBC = perceived behavioral control, BI = behavioral intention, S.E. = standard error. Note 2: *** *p* < 0.001.

## Data Availability

The data supporting the findings of this study are not publicly available because the dataset includes personal data from participants who consented under the conditions of confidentiality and non-disclosure, and we are obliged to uphold privacy rights. However, the data are available from the corresponding author, H.-S.C., upon reasonable request, and with respect to the aforementioned constraints.
